# RETRACTION: Effect of Single‐Port Video‐Assisted Thoracoscopy on Surgical Site Wound Infection and Healing in Patients With Lung Cancer: A Meta‐Analysis

**DOI:** 10.1111/iwj.70300

**Published:** 2025-03-06

## Abstract

**RETRACTION:** WuW. , ZhaoY. , ZhangZ. , JiangJ. , FengC. , QinD. , ZhangC. , XuZ. , ZhangL. , and LinF. , “Effect of Single‐Port Video‐Assisted Thoracoscopy on Surgical Site Wound Infection and Healing in Patients with Lung Cancer: A Meta‐Analysis,” International Wound Journal 20, no. 9 (2023): 3483–3490, 10.1111/iwj.14220.37193587 PMC10588324

The above article, published online on 16 May 2023, in Wiley Online Library (http://onlinelibrary.wiley.com/), has been retracted by agreement between the journal Editor in Chief, Professor Keith Harding; and John Wiley & Sons Ltd. Following an investigation by the publisher, all parties have concluded that this article was accepted solely on the basis of a compromised peer review process. The editors have therefore decided to retract the article. The authors did not indicate their agreement with the retraction and made a statement that they were not responsible for the compromised peer review.

**RETRACTION:** W. Wu , Y. Zhao , Z. Zhang , J. Jiang , C. Feng , D. Qin , C. Zhang , Z. Xu , L. Zhang , and F. Lin , “Effect of Single‐Port Video‐Assisted Thoracoscopy on Surgical Site Wound Infection and Healing in Patients with Lung Cancer: A Meta‐Analysis,” International Wound Journal 20, no. 9 (2023): 3483–3490, 10.1111/iwj.14220.37193587 PMC10588324

The above article, published online on 16 May 2023, in Wiley Online Library (http://onlinelibrary.wiley.com/), has been retracted by agreement between the journal Editor in Chief, Professor Keith Harding; and John Wiley & Sons Ltd. Following an investigation by the publisher, all parties have concluded that this article was accepted solely on the basis of a compromised peer review process. The editors have therefore decided to retract the article. The authors did not indicate their agreement with the retraction and made a statement that they were not responsible for the compromised peer review.

